# Supplementary material for: Perceptions, behaviours and barriers towards exercise practices in inflammatory bowel disease
Source: PLoS One. 2024 Apr 5;19(4):e0299228. doi: 10.1371/journal.pone.0299228 (PMC10997097; doi:10.1371/journal.pone.0299228)
Supplement: S1 Appendix — (DOCX) [file pone.0299228.s001.docx]

**Supplementary materials 1 (percentages are calculated for each table row)**

**Appendix A: Do you exercise regularly? —two-way cross-tabulation expressed as a function of Gender**

|  | ***Do you exercise regularly?*** | | | |
| --- | --- | --- | --- | --- |
|  | Yes | | No | |
| ***Gender*** | **N** | **%** | **N** | **%** |
| Male | 77 | 74.04 | 27 | 25.96 |
| Female | 212 | 59.72 | 143 | 40.28 |
| Non-binary | 3 | 75.00 | 1 | 25.00 |

**Appendix B: Which type of resistance exercise to you mostly do? —two-way cross-tabulation expressed as a function of Gender**

|  | ***Which type of resistance exercise to you mostly do?*** | | | | | | | | | | | | |
| --- | --- | --- | --- | --- | --- | --- | --- | --- | --- | --- | --- | --- | --- |
|  | None | | Bodyweight | | Structured classes | | Machines | | Free weights | | Other | |  |
| ***Gender*** | **N** | **%** | **N** | **%** | **N** | **%** | **N** | **%** | **N** | **%** | **N** | **%** |  |
| Male | 19 | 24.68 | 17 | 22.08 | 2 | 2.60 | 18 | 23.38 | 18 | 23.38 | 3 | 3.90 |  |
| Female | 62 | 29.25 | 56 | 26.42 | 19 | 8.96 | 16 | 7.55 | 39 | 18.40 | 20 | 9.43 |  |
| Non-binary | 0 | 0.00 | 0 | 0.00 | 0 | 0.00 | 0 | 0.00 | 2 | 66.67 | 1 | 33.33 |  |

**Appendix C: Do you avoid certain types of exercise? —two-way cross-tabulation expressed as a function of Gender**

|  | ***Do you avoid certain types of exercise?*** | | | | | | | | | | | | | | | | | | | | |
| --- | --- | --- | --- | --- | --- | --- | --- | --- | --- | --- | --- | --- | --- | --- | --- | --- | --- | --- | --- | --- | --- |
|  | No | | Low intensity | | Moderate intensity | | Vigorous intensity | | Bodyweight exercise | | Structured weights classes | | Free weights | | | Machine based weightlifting | | Other | | Combination | |
| ***Gender*** | **N** | **%** | **N** | **%** | **N** | **%** | **N** | **%** | **N** | **%** | **N** | **%** | **N** |  | **%** | **N** | **%** | **N** | **%** | **N** | **%** |
| Male | 33 | 42.86 | 0 | 0.00 | 0 | 0.00 | 4 | 5.19 | 1 | 1.30 | 2 | 2.60 | 5 |  | 6.49 | 0 | 0.00 | 3 | 3.90 | 29 | 37.66 |
| Female | 98 | 46.23 | 3 | 1.42 | 2 | 0.94 | 27 | 12.74 | 1 | 0.47 | 4 | 1.89 | 0 |  | 0.00 | 3 | 1.42 | 13 | 6.13 | 61 | 28.77 |
| Non-binary | 1 | 33.33 | 1 | 33.33 | 0 | 0.00 | 0 | 0.00 | 0 | 0.00 | 0 | 0.00 | 0 |  | 0.00 | 0 | 0.00 | 0 | 0.00 | 1 | 33.33 |

**Appendix D: Which type of aerobic exercise to you mostly do? —two-way cross-tabulation expressed as a function of Disease activity**

|  | ***Which type of aerobic exercise to you mostly do?*** | | | | | | | |
| --- | --- | --- | --- | --- | --- | --- | --- | --- |
|  | None | | Low intensity | | Moderate intensity | | Vigorous intensity | |
| ***Disease activity*** | **N** | **%** | **N** | **%** | **N** | **%** | **N** | **%** |
| Remission | 3 | 3.09 | 26 | 26.80 | 42 | 43.30 | 26 | 26.80 |
| Mild | 1 | 0.90 | 39 | 35.14 | 47 | 42.34 | 24 | 21.62 |
| Moderate | 3 | 4.23 | 29 | 40.85 | 22 | 30.99 | 17 | 23.94 |
| Severe | 3 | 23.08 | 2 | 15.38 | 3 | 23.08 | 5 | 38.46 |

**Appendix E: What would/ does prevent you from engaging in exercise? —two-way cross-tabulation expressed as a function of Disease activity**

|  | ***What would/ does prevent you from engaging in exercise?*** | | | | | | | | | | | | | | | |
| --- | --- | --- | --- | --- | --- | --- | --- | --- | --- | --- | --- | --- | --- | --- | --- | --- |
|  | Fear of increased toilet urgency | | Fear of increased abdominal pain | | Fear of triggering a flare up | | Lack of scientific evidence | | Fatigue | | Pain during exercise | | Other | | Combination of factors | |
| ***Disease activity*** | **N** | **%** | **N** | **%** | **N** | **%** | **N** | **%** | **N** | **%** | **N** | **%** | **N** | **%** | **N** | **%** |
| Remission | 8 | 5.67 | 1 | 0.71 | 1 | 0.71 | 1 | 0.71 | 24 | 17.02 | 1 | 0.71 | 13 | 9.22 | 92 | 65.25 |
| Mild | 7 | 4.14 | 0 | 0.00 | 0 | 0.00 | 0 | 0.00 | 26 | 15.38 | 0 | 0.00 | 6 | 3.55 | 130 | 76.92 |
| Moderate | 10 | 7.75 | 0 | 0.00 | 1 | 0.78 | 0 | 0.00 | 11 | 8.53 | 1 | 0.78 | 1 | 0.78 | 105 | 81.40 |
| Severe | 3 | 12.50 | 0 | 0.00 | 0 | 0.00 | 0 | 0.00 | 3 | 12.50 | 1 | 4.17 | 0 | 0.00 | 17 | 70.83 |
