# Supplementary material for: Perceptions, behaviours and barriers towards exercise practices in inflammatory bowel disease
Source: PLoS One. 2024 Apr 5;19(4):e0299228. doi: 10.1371/journal.pone.0299228 (PMC10997097; doi:10.1371/journal.pone.0299228)
Supplement: S2 Appendix — (DOCX) [file pone.0299228.s002.docx]

**Supplementary materials 2 (percentages are calculated for each table row)**

**Age**

|  | ***Do you exercise regularly?*** | | | |
| --- | --- | --- | --- | --- |
|  | Yes | | No | |
| ***Age*** | **N** | **%** | **N** | **%** |
| 18-25 | 31 | 68.89 | 14 | 31.11 |
| 26-30 | 28 | 63.64 | 16 | 36.36 |
| 31-35 | 37 | 62.71 | 22 | 37.29 |
| 36-40 | 52 | 61.90 | 32 | 38.10 |
| 41-45 | 39 | 60.00 | 26 | 40.00 |
| 46-50 | 44 | 66.67 | 22 | 33.33 |
| 51-55 | 31 | 68.89 | 14 | 31.11 |
| 56-60 | 11 | 45.83 | 13 | 54.17 |
| 61-65 | 12 | 54.55 | 10 | 45.45 |
| 65+ | 7 | 77.78 | 2 | 22.22 |

|  | ***Which type of aerobic exercise do you mostly do?*** | | | | | | |  |
| --- | --- | --- | --- | --- | --- | --- | --- | --- |
|  | None | | Low intensity | | Moderate intensity | | Vigorous | |
| ***Age*** | **N** | **%** | **N** | **%** | **N** | **%** | **N** | **%** |
| 18-25 | 1 | 3.23 | 4 | 12.90 | 18 | 58.06 | 8 | 25.81 |
| 26-30 | 0 | 0.00 | 9 | 32.14 | 9 | 32.14 | 10 | 35.71 |
| 31-35 | 2 | 5.41 | 7 | 18.92 | 14 | 37.84 | 14 | 37.84 |
| 36-40 | 0 | 0.00 | 27 | 51.92 | 16 | 30.77 | 9 | 17.31 |
| 41-45 | 0 | 0.00 | 13 | 33.33 | 15 | 38.46 | 11 | 28.21 |
| 46-50 | 2 | 4.55 | 13 | 29.55 | 19 | 43.18 | 10 | 22.73 |
| 51-55 | 3 | 9.68 | 9 | 29.03 | 13 | 41.94 | 6 | 19.35 |
| 56-60 | 0 | 0.00 | 5 | 45.45 | 4 | 36.36 | 2 | 18.18 |
| 61-65 | 1 | 8.33 | 6 | 50.00 | 4 | 33.33 | 1 | 8.33 |
| 65+ | 1 | 14.29 | 3 | 42.86 | 2 | 28.57 | 1 | 14.29 |

|  | ***Do you believe aerobic exercise can influence Inflammatory bowel dis-ease in a positive way?*** | | | | | |
| --- | --- | --- | --- | --- | --- | --- |
|  | Yes | | No | | Don't know | |
| ***Age*** | **N** | **%** | **N** | **%** | **N** | **%** |
| 18-25 | 17 | 54.84 | 1 | 3.23 | 13 | 41.94 |
| 26-30 | 17 | 60.71 | 1 | 3.57 | 10 | 35.71 |
| 31-35 | 22 | 59.46 | 1 | 2.70 | 14 | 37.84 |
| 36-40 | 35 | 67.31 | 3 | 5.77 | 14 | 26.92 |
| 41-45 | 27 | 69.23 | 0 | 0.00 | 12 | 30.77 |
| 46-50 | 23 | 52.27 | 1 | 2.27 | 20 | 45.45 |
| 51-55 | 14 | 45.16 | 5 | 16.13 | 12 | 38.71 |
| 56-60 | 4 | 36.36 | 0 | 0.00 | 7 | 63.64 |
| 61-65 | 5 | 41.67 | 1 | 8.33 | 6 | 50.00 |
| 65+ | 4 | 57.14 | 0 | 0.00 | 3 | 42.86 |

|  | ***Do you believe aerobic exercise can influence Inflammatory bowel dis-ease in a negative way?*** | | | | | |
| --- | --- | --- | --- | --- | --- | --- |
|  | Yes | | No | | Don't know | |
| ***Age*** | **N** | **%** | **N** | **%** | **N** | **%** |
| 18-25 | 13 | 41.94 | 6 | 19.35 | 12 | 38.71 |
| 26-30 | 8 | 28.57 | 7 | 25.00 | 13 | 46.43 |
| 31-35 | 12 | 32.43 | 12 | 32.43 | 13 | 35.14 |
| 36-40 | 11 | 21.15 | 17 | 32.69 | 24 | 46.15 |
| 41-45 | 12 | 30.77 | 13 | 33.33 | 14 | 35.90 |
| 46-50 | 18 | 40.91 | 12 | 27.27 | 14 | 31.82 |
| 51-55 | 8 | 25.81 | 12 | 38.71 | 11 | 35.48 |
| 56-60 | 2 | 18.18 | 0 | 0.00 | 9 | 81.82 |
| 61-65 | 2 | 16.67 | 4 | 33.33 | 6 | 50.00 |
| 65+ | 1 | 14.29 | 3 | 42.86 | 3 | 42.86 |

|  | ***Which type of resistance exercise to you mostly do?*** | | | | | | | | | |  |  |  |
| --- | --- | --- | --- | --- | --- | --- | --- | --- | --- | --- | --- | --- | --- |
|  | None | | Bodyweight | | Structured classes | | Machines | | Free weights | | | Other | |
| ***Age*** | **N** | **%** | **N** | **%** | **N** | **%** | **N** | **%** | **N** | **%** | | **N** | **%** |
| 18-25 | 8 | 25.81 | 9 | 29.03 | 3 | 9.68 | 3 | 9.68 | 7 | 22.58 | | 1 | 3.23 |
| 26-30 | 4 | 14.29 | 7 | 25.00 | 0 | 0.00 | 6 | 21.43 | 7 | 25.00 | | 4 | 14.29 |
| 31-35 | 8 | 21.62 | 8 | 21.62 | 5 | 13.51 | 4 | 10.81 | 9 | 24.32 | | 3 | 8.11 |
| 36-40 | 15 | 28.85 | 9 | 17.31 | 3 | 5.77 | 10 | 19.23 | 10 | 19.23 | | 5 | 9.62 |
| 41-45 | 11 | 28.21 | 11 | 28.21 | 2 | 5.13 | 3 | 7.69 | 10 | 25.64 | | 2 | 5.13 |
| 46-50 | 15 | 34.09 | 14 | 31.82 | 3 | 6.82 | 3 | 6.82 | 7 | 15.91 | | 2 | 4.55 |
| 51-55 | 10 | 32.26 | 8 | 25.81 | 2 | 6.45 | 3 | 9.68 | 4 | 12.90 | | 4 | 12.90 |
| 56-60 | 6 | 54.55 | 1 | 9.09 | 0 | 0.00 | 1 | 9.09 | 2 | 18.18 | | 1 | 9.09 |
| 61-65 | 3 | 25.00 | 3 | 25.00 | 2 | 16.67 | 0 | 0.00 | 2 | 16.67 | | 2 | 16.67 |
| 65+ | 1 | 14.29 | 3 | 42.86 | 1 | 14.29 | 1 | 14.29 | 1 | 14.29 | | 0 | 0.00 |

|  | ***Do you believe resistance exercise can influence Inflammatory bowel disease in a positive way?*** | | | | | |
| --- | --- | --- | --- | --- | --- | --- |
|  | Yes | | No | | Don't know | |
| ***Age*** | **N** | **%** | **N** | **%** | **N** | **%** |
| 18-25 | 6 | 19.35 | 0 | 0.00 | 25 | 80.65 |
| 26-30 | 12 | 42.86 | 2 | 7.14 | 14 | 50.00 |
| 31-35 | 22 | 59.46 | 0 | 0.00 | 15 | 40.54 |
| 36-40 | 21 | 40.38 | 3 | 5.77 | 28 | 53.85 |
| 41-45 | 14 | 35.90 | 4 | 10.26 | 21 | 53.85 |
| 46-50 | 16 | 36.36 | 2 | 4.55 | 26 | 59.09 |
| 51-55 | 10 | 32.26 | 2 | 6.45 | 19 | 61.29 |
| 56-60 | 1 | 9.09 | 0 | 0.00 | 10 | 90.91 |
| 61-65 | 5 | 41.67 | 1 | 8.33 | 6 | 50.00 |
| 65+ | 3 | 42.86 | 0 | 0.00 | 4 | 57.14 |

|  | ***Do you believe resistance exercise can influence Inflammatory bowel disease in a negative way?*** | | | | | |
| --- | --- | --- | --- | --- | --- | --- |
|  | Yes | | No | | Don't know | |
| ***Age*** | **N** | **%** | **N** | **%** | **N** | **%** |
| 18-25 | 4 | 12.90 | 6 | 19.35 | 21 | 67.74 |
| 26-30 | 0 | 0.00 | 12 | 42.86 | 16 | 57.14 |
| 31-35 | 3 | 8.11 | 9 | 24.32 | 25 | 67.57 |
| 36-40 | 4 | 7.69 | 15 | 28.85 | 33 | 63.46 |
| 41-45 | 5 | 12.82 | 11 | 28.21 | 23 | 58.97 |
| 46-50 | 4 | 9.09 | 11 | 25.00 | 29 | 65.91 |
| 51-55 | 2 | 6.45 | 10 | 32.26 | 19 | 61.29 |
| 56-60 | 1 | 9.09 | 1 | 9.09 | 9 | 81.82 |
| 61-65 | 2 | 16.67 | 5 | 41.67 | 5 | 41.67 |
| 65+ | 1 | 14.29 | 4 | 57.14 | 2 | 28.57 |

|  | ***Do you avoid certain types of exercise?*** | | | | | | | | | | | | | | | | | | | |
| --- | --- | --- | --- | --- | --- | --- | --- | --- | --- | --- | --- | --- | --- | --- | --- | --- | --- | --- | --- | --- |
|  | No | | Low intensity | | Moderate intensity | | Vigorous intensity | | Bodyweight exercise | | Structured weights classes | | Free weights | | Machine based weightlifting | | Other | | Combination | |
| ***Age*** | **N** | **%** | **N** | **%** | **N** | **%** | **N** | **%** | **N** | **%** | **N** | **%** | **N** | **%** | **N** | **%** | **N** | **%** | **N** | **%** |
| 18-25 | 18 | 58.06 | 0 | 0.00 | 0 | 0.00 | 4 | 12.90 | 0 | 0.00 | 0 | 0.00 | 0 | 0.00 | 1 | 3.23 | 2 | 6.45 | 6 | 19.35 |
| 26-30 | 13 | 46.43 | 0 | 0.00 | 0 | 0.00 | 3 | 10.71 | 0 | 0.00 | 1 | 3.57 | 0 | 0.00 | 0 | 0.00 | 3 | 10.71 | 8 | 28.57 |
| 31-35 | 17 | 45.95 | 1 | 2.70 | 1 | 2.70 | 4 | 10.81 | 0 | 0.00 | 0 | 0.00 | 1 | 2.70 | 0 | 0.00 | 2 | 5.41 | 11 | 29.73 |
| 36-40 | 19 | 36.54 | 0 | 0.00 | 0 | 0.00 | 11 | 21.15 | 0 | 0.00 | 1 | 1.92 | 4 | 7.69 | 0 | 0.00 | 3 | 5.77 | 14 | 26.92 |
| 41-45 | 20 | 51.28 | 0 | 0.00 | 0 | 0.00 | 4 | 10.26 | 0 | 0.00 | 2 | 5.13 | 0 | 0.00 | 0 | 0.00 | 4 | 10.26 | 9 | 23.08 |
| 46-50 | 20 | 45.45 | 2 | 4.55 | 0 | 0.00 | 4 | 9.09 | 2 | 4.55 | 0 | 0.00 | 0 | 0.00 | 1 | 2.27 | 1 | 2.27 | 14 | 31.82 |
| 51-55 | 16 | 51.61 | 0 | 0.00 | 1 | 3.23 | 0 | 0.00 | 0 | 0.00 | 0 | 0.00 | 0 | 0.00 | 0 | 0.00 | 0 | 0.00 | 14 | 45.16 |
| 56-60 | 4 | 36.36 | 1 | 9.09 | 0 | 0.00 | 0 | 0.00 | 0 | 0.00 | 1 | 9.09 | 0 | 0.00 | 0 | 0.00 | 0 | 0.00 | 5 | 45.45 |
| 61-65 | 3 | 25.00 | 0 | 0.00 | 0 | 0.00 | 1 | 8.33 | 0 | 0.00 | 1 | 8.33 | 0 | 0.00 | 1 | 8.33 | 1 | 8.33 | 5 | 41.67 |
| 65+ | 2 | 28.57 | 0 | 0.00 | 0 | 0.00 | 0 | 0.00 | 0 | 0.00 | 0 | 0.00 | 0 | 0.00 | 0 | 0.00 | 0 | 0.00 | 5 | 71.43 |

|  | ***What would/ does prevent you from engaging in exercise?*** | | | | | | | | | | | | | | | |
| --- | --- | --- | --- | --- | --- | --- | --- | --- | --- | --- | --- | --- | --- | --- | --- | --- |
|  | Fear of increased toilet urgency | | Fear of increased abdominal pain | | Fear of triggering a flare up | | Lack of scientific evidence | | Fatigue | | Pain during exercise | | Other | | Combination of factors | |
| ***Age*** | **N** | **%** | **N** | **%** | **N** | **%** | **N** | **%** | **N** | **%** | **N** | **%** | **N** | **%** | **N** | **%** |
| 18-25 | 2 | 4.44 | 1 | 2.22 | 0 | 0.00 | 0 | 0.00 | 8 | 17.78 | 0 | 0.00 | 0 | 0.00 | 34 | 75.56 |
| 26-30 | 3 | 6.82 | 0 | 0.00 | 0 | 0.00 | 0 | 0.00 | 4 | 9.09 | 0 | 0.00 | 4 | 9.09 | 33 | 75.00 |
| 31-35 | 2 | 3.39 | 0 | 0.00 | 0 | 0.00 | 0 | 0.00 | 11 | 18.64 | 0 | 0.00 | 3 | 5.08 | 43 | 72.88 |
| 36-40 | 2 | 2.38 | 0 | 0.00 | 0 | 0.00 | 0 | 0.00 | 12 | 14.29 | 0 | 0.00 | 3 | 3.57 | 67 | 79.76 |
| 41-45 | 5 | 7.69 | 0 | 0.00 | 1 | 1.54 | 0 | 0.00 | 2 | 3.08 | 0 | 0.00 | 3 | 4.62 | 54 | 83.08 |
| 46-50 | 5 | 7.58 | 0 | 0.00 | 0 | 0.00 | 0 | 0.00 | 10 | 15.15 | 1 | 1.52 | 2 | 3.03 | 48 | 72.73 |
| 51-55 | 5 | 11.11 | 0 | 0.00 | 1 | 2.22 | 0 | 0.00 | 10 | 22.22 | 0 | 0.00 | 2 | 4.44 | 27 | 60.00 |
| 56-60 | 1 | 4.17 | 0 | 0.00 | 0 | 0.00 | 0 | 0.00 | 4 | 16.67 | 0 | 0.00 | 2 | 8.33 | 17 | 70.83 |
| 61-65 | 1 | 4.55 | 0 | 0.00 | 0 | 0.00 | 1 | 4.55 | 3 | 13.64 | 1 | 4.55 | 1 | 4.55 | 15 | 68.18 |
| 65+ | 2 | 22.22 | 0 | 0.00 | 0 | 0.00 | 0 | 0.00 | 0 | 0.00 | 1 | 11.11 | 0 | 0.00 | 6 | 66.67 |

|  | ***Would you consider undertaking whole body vibration exercise?*** | | | |
| --- | --- | --- | --- | --- |
|  | Yes | | No | |
| ***Age*** | **N** | **%** | **N** | **%** |
| 18-25 | 39 | 86.67 | 6 | 13.33 |
| 26-30 | 40 | 90.91 | 4 | 9.09 |
| 31-35 | 56 | 94.92 | 3 | 5.08 |
| 36-40 | 82 | 97.62 | 2 | 2.38 |
| 41-45 | 54 | 83.08 | 11 | 16.92 |
| 46-50 | 62 | 93.94 | 4 | 6.06 |
| 51-55 | 43 | 95.56 | 2 | 4.44 |
| 56-60 | 24 | 100.00 | 0 | 0.00 |
| 61-65 | 21 | 95.45 | 1 | 4.55 |
| 65+ | 7 | 77.78 | 2 | 22.22 |

**Gender**

|  | ***Which type of aerobic exercise do you mostly do?*** | | | | | | | |
| --- | --- | --- | --- | --- | --- | --- | --- | --- |
|  | None | | Low intensity | | Moderate intensity | | Vigorous | |
| ***Gender*** | **N** | **%** | **N** | **%** | **N** | **%** | **N** | **%** |
| Male | 3 | 3.90 | 29 | 37.66 | 23 | 29.87 | 22 | 28.57 |
| Female | 7 | 3.30 | 67 | 31.60 | 89 | 41.98 | 49 | 23.11 |
| Non-binary | 0 | 0.00 | 0 | 0.00 | 2 | 66.67 | 1 | 33.33 |

|  | ***Do you believe aerobic exercise can influence Inflammatory bowel dis-ease in a positive way?*** | | | | | |
| --- | --- | --- | --- | --- | --- | --- |
|  | Yes | | No | | Don't know | |
| ***Gender*** | **N** | **%** | **N** | **%** | **N** | **%** |
| Male | 54 | 70.13 | 2 | 2.60 | 21 | 27.27 |
| Female | 113 | 53.30 | 11 | 5.19 | 88 | 41.51 |
| Non-binary | 1 | 33.33 | 0 | 0.00 | 2 | 66.67 |

|  | ***Do you believe aerobic exercise can influence Inflammatory bowel dis-ease in a negative way?*** | | | | | |
| --- | --- | --- | --- | --- | --- | --- |
|  | Yes | | No | | Don't know | |
| ***Gender*** | **N** | **%** | **N** | **%** | **N** | **%** |
| Male | 17 | 22.08 | 23 | 29.87 | 37 | 48.05 |
| Female | 68 | 32.08 | 63 | 29.72 | 81 | 38.21 |
| Non-binary | 2 | 66.67 | 0 | 0.00 | 1 | 33.33 |

|  | ***Do you believe resistance exercise can influence Inflammatory bowel disease in a positive way?*** | | | | | |
| --- | --- | --- | --- | --- | --- | --- |
|  | Yes | | No | | Don't know | |
| ***Gender*** | **N** | **%** | **N** | **%** | **N** | **%** |
| Male | 33 | 42.86 | 3 | 3.90 | 41 | 53.25 |
| Female | 77 | 36.32 | 11 | 5.19 | 124 | 58.49 |
| Non-binary | 0 | 0.00 | 0 | 0.00 | 3 | 100.00 |

|  | ***Do you believe resistance exercise can influence Inflammatory bowel dis-ease in a negative way?*** | | | | | |
| --- | --- | --- | --- | --- | --- | --- |
|  | Yes | | No | | Don't know | |
| ***Gender*** | **N** | **%** | **N** | **%** | **N** | **%** |
| Male | 8 | 10.39 | 19 | 24.68 | 50 | 64.94 |
| Female | 18 | 8.49 | 65 | 30.66 | 129 | 60.85 |
| Non-binary | 0 | 0.00 | 0 | 0.00 | 3 | 100.00 |

|  | ***What would/ does prevent you from engaging in exercise?*** | | | | | | | | | | | | | | | |
| --- | --- | --- | --- | --- | --- | --- | --- | --- | --- | --- | --- | --- | --- | --- | --- | --- |
|  | Fear of increased toilet urgency | | Fear of increased abdominal pain | | Fear of triggering a flare up | | Lack of scientific evidence | | Fatigue | | Pain during exercise | | Other | | Combination of factors | |
| ***Gender*** | **N** | **%** | **N** | **%** | **N** | **%** | **N** | **%** | **N** | **%** | **N** | **%** | **N** | **%** | **N** | **%** |
| Male | 7 | 6.73 | 1 | 0.96 | 1 | 0.96 | 0 | 0.00 | 10 | 9.62 | 1 | 0.96 | 7 | 6.73 | 77 | 74.04 |
| Female | 21 | 5.92 | 0 | 0.00 | 1 | 0.28 | 1 | 0.28 | 54 | 15.21 | 2 | 0.56 | 13 | 3.66 | 263 | 74.08 |
| Non-binary | 0 | 0.00 | 0 | 0.00 | 0 | 0.00 | 0 | 0.00 | 0 | 0.00 | 0 | 0.00 | 0 | 0.00 | 4 | 100.00 |

|  | ***Would you consider undertaking whole body vibration exercise?*** | | | |
| --- | --- | --- | --- | --- |
|  | Yes | | No | |
| ***Gender*** | **N** | **%** | **N** | **%** |
| Male | 95 | 91.35 | 9 | 8.65 |
| Female | 330 | 92.96 | 25 | 7.04 |
| Non-binary | 3 | 75.00 | 1 | 25.00 |

**Disease**

|  | ***Do you exercise regularly?*** | | | |
| --- | --- | --- | --- | --- |
|  | Yes | | No | |
| ***Disease*** | **N** | **%** | **N** | **%** |
| UC | 165 | 62.50 | 99 | 37.50 |
| CD | 120 | 63.83 | 68 | 36.17 |
| Other | 7 | 63.64 | 4 | 36.36 |

|  | ***Which type of aerobic exercise do you mostly do?*** | | | | | | | |
| --- | --- | --- | --- | --- | --- | --- | --- | --- |
|  | None | | Low intensity | | Moderate intensity | | Vigorous | |
| ***Disease*** | **N** | **%** | **N** | **%** | **N** | **%** | **N** | **%** |
| UC | 7 | 4.24 | 59 | 35.76 | 62 | 37.58 | 37 | 22.42 |
| CD | 2 | 1.67 | 36 | 30.00 | 50 | 41.67 | 32 | 26.67 |
| Other | 1 | 14.29 | 1 | 14.29 | 2 | 28.57 | 3 | 42.86 |

|  | ***Do you believe aerobic exercise can influence Inflammatory bowel dis-ease in a positive way?*** | | | | | |
| --- | --- | --- | --- | --- | --- | --- |
|  | Yes | | No | | Don't know | |
| ***Disease*** | **N** | **%** | **N** | **%** | **N** | **%** |
| UC | 98 | 59.39 | 10 | 6.06 | 57 | 34.55 |
| CD | 68 | 56.67 | 3 | 2.50 | 49 | 40.83 |
| Other | 2 | 28.57 | 0 | 0.00 | 5 | 71.43 |

|  | ***Do you believe aerobic exercise can influence Inflammatory bowel dis-ease in a negative way?*** | | | | | |
| --- | --- | --- | --- | --- | --- | --- |
|  | Yes | | No | | Don't know | |
| ***Disease*** | **N** | **%** | **N** | **%** | **N** | **%** |
| UC | 45 | 27.27 | 51 | 30.91 | 69 | 41.82 |
| CD | 39 | 32.50 | 33 | 27.50 | 48 | 40.00 |
| Other | 3 | 42.86 | 2 | 28.57 | 2 | 28.57 |

|  | ***Which type of resistance exercise to you mostly do?*** | | | | | | | | | |  |  |  |  |
| --- | --- | --- | --- | --- | --- | --- | --- | --- | --- | --- | --- | --- | --- | --- |
|  | None | | Bodyweight | | Structured classes | | Machines | | Free weights | | | Other | | |
| ***Disease*** | **N** | **%** | **N** | **%** | **N** | **%** | **N** | **%** | **N** | **%** | | **N** | **%** | |
| UC | 52 | 31.52 | 43 | 26.06 | 10 | 6.06 | 19 | 11.52 | 28 | 16.97 | | 13 | 7.88 | |
| CD | 28 | 23.33 | 27 | 22.50 | 10 | 8.33 | 15 | 12.50 | 29 | 24.17 | | 11 | 9.17 | |
| Other | 1 | 14.29 | 3 | 42.86 | 1 | 14.29 | 0 | 0.00 | 2 | 28.57 | | 0 | 0.00 | |

|  | ***Do you believe resistance exercise can influence Inflammatory bowel disease in a positive way?*** | | | | | |
| --- | --- | --- | --- | --- | --- | --- |
|  | Yes | | No | | Don't know | |
| ***Disease*** | **N** | **%** | **N** | **%** | **N** | **%** |
| UC | 69 | 41.82 | 10 | 6.06 | 86 | 52.12 |
| CD | 40 | 33.33 | 4 | 3.33 | 76 | 63.33 |
| Other | 1 | 14.29 | 0 | 0.00 | 6 | 85.71 |

|  | ***Do you believe resistance exercise can influence Inflammatory bowel dis-ease in a negative way?*** | | | | | |
| --- | --- | --- | --- | --- | --- | --- |
|  | Yes | | No | | Don't know | |
| ***Disease*** | **N** | **%** | **N** | **%** | **N** | **%** |
| UC | 15 | 9.09 | 54 | 32.73 | 96 | 58.18 |
| CD | 11 | 9.17 | 29 | 24.17 | 80 | 66.67 |
| Other | 0 | 0.00 | 1 | 14.29 | 6 | 85.71 |

|  | ***Do you avoid certain types of exercise?*** | | | | | | | | | | | | | | | | | | | |
| --- | --- | --- | --- | --- | --- | --- | --- | --- | --- | --- | --- | --- | --- | --- | --- | --- | --- | --- | --- | --- |
|  | No | | Low intensity | | Moderate intensity | | Vigorous intensity | | Bodyweight exercise | | Structured weights classes | | Free weights | | Machine based weightlifting | | Other | | Combination | |
| ***Disease*** | **N** | **%** | **N** | **%** | **N** | **%** | **N** | **%** | **N** | **%** | **N** | **%** | **N** | **%** | **N** | **%** | **N** | **%** | **N** | **%** |
| UC | 79 | 47.88 | 1 | 0.61 | 0 | 0.00 | 20 | 12.12 | 0 | 0.00 | 2 | 1.21 | 1 | 0.61 | 0 | 0.00 | 9 | 5.45 | 53 | 32.12 |
| CD | 50 | 41.67 | 2 | 1.67 | 2 | 1.67 | 11 | 9.17 | 2 | 1.67 | 4 | 3.33 | 4 | 3.33 | 3 | 2.50 | 5 | 4.17 | 37 | 30.83 |
| Other | 3 | 42.86 | 1 | 14.29 | 0 | 0.00 | 0 | 0.00 | 0 | 0.00 | 0 | 0.00 | 0 | 0.00 | 0 | 0.00 | 2 | 28.57 | 1 | 14.29 |

|  | ***What would/ does prevent you from engaging in exercise?*** | | | | | | | | | | | | | | | |
| --- | --- | --- | --- | --- | --- | --- | --- | --- | --- | --- | --- | --- | --- | --- | --- | --- |
|  | Fear of increased toilet urgency | | Fear of increased abdominal pain | | Fear of triggering a flare up | | Lack of scientific evidence | | Fatigue | | Pain during exercise | | Other | | Combination of factors | |
| ***Disease*** | **N** | **%** | **N** | **%** | **N** | **%** | **N** | **%** | **N** | **%** | **N** | **%** | **N** | **%** | **N** | **%** |
| UC | 12 | 4.55 | 0 | 0.00 | 2 | 0.76 | 1 | 0.38 | 44 | 16.67 | 1 | 0.38 | 10 | 3.79 | 194 | 73.48 |
| CD | 15 | 7.98 | 1 | 0.53 | 0 | 0.00 | 0 | 0.00 | 19 | 10.11 | 1 | 0.53 | 10 | 5.32 | 142 | 75.53 |
| Other | 1 | 9.09 | 0 | 0.00 | 0 | 0.00 | 0 | 0.00 | 1 | 9.09 | 1 | 9.09 | 0 | 0.00 | 8 | 72.73 |

|  | ***Would you consider undertaking whole body vibration exercise?*** | | | |
| --- | --- | --- | --- | --- |
|  | Yes | | No | |
| ***Disease*** | **N** | **%** | **N** | **%** |
| UC | 242 | 91.67 | 22 | 8.33 |
| CD | 177 | 94.15 | 11 | 5.85 |
| Other | 9 | 81.82 | 2 | 18.18 |

**Disease activity**

|  | ***Do you exercise regularly?*** | | | |
| --- | --- | --- | --- | --- |
|  | Yes | | No | |
| ***Disease activity*** | **N** | **%** | **N** | **%** |
| Remission | 97 | 68.79 | 44 | 31.21 |
| Mild | 111 | 65.68 | 58 | 34.32 |
| Moderate | 71 | 55.04 | 58 | 44.96 |
| Severe | 13 | 54.17 | 11 | 45.83 |

|  | ***Do you believe aerobic exercise can influence Inflammatory bowel disease in a positive way?*** | | | | | |
| --- | --- | --- | --- | --- | --- | --- |
|  | Yes | | No | | Don't know | |
| ***Disease activity*** | **N** | **%** | **N** | **%** | **N** | **%** |
| Remission | 63 | 64.95 | 4 | 4.12 | 30 | 30.93 |
| Mild | 65 | 58.56 | 3 | 2.70 | 43 | 38.74 |
| Moderate | 32 | 45.07 | 5 | 7.04 | 34 | 47.89 |
| Severe | 8 | 61.54 | 1 | 7.69 | 4 | 30.77 |

|  | ***Do you believe aerobic exercise can influence Inflammatory bowel disease in a negative way?*** | | | | | |
| --- | --- | --- | --- | --- | --- | --- |
|  | Yes | | No | | Don't know | |
| ***Disease activity*** | **N** | **%** | **N** | **%** | **N** | **%** |
| Remission | 25 | 25.77 | 38 | 39.18 | 34 | 35.05 |
| Mild | 31 | 27.93 | 29 | 26.13 | 51 | 45.95 |
| Moderate | 26 | 36.62 | 15 | 21.13 | 30 | 42.25 |
| Severe | 5 | 38.46 | 4 | 30.77 | 4 | 30.77 |

|  | ***Which type of resistance exercise to you mostly do?*** | | | | | | | | |  |  |  |
| --- | --- | --- | --- | --- | --- | --- | --- | --- | --- | --- | --- | --- |
|  | None | | Bodyweight | | Structured classes | | Machines | | Free weights | | Other | |
| ***Disease activity*** | **N** | **%** | **N** | **%** | **N** | **%** | **N** | **%** | **N** | **%** | **N** | **%** |
| Remission | 26 | 26.80 | 31 | 31.96 | 5 | 5.15 | 11 | 11.34 | 18 | 18.56 | 6 | 6.19 |
| Mild | 27 | 24.32 | 26 | 23.42 | 9 | 8.11 | 18 | 16.22 | 24 | 21.62 | 7 | 6.31 |
| Moderate | 22 | 30.99 | 14 | 19.72 | 6 | 8.45 | 5 | 7.04 | 14 | 19.72 | 10 | 14.08 |
| Severe | 6 | 46.15 | 2 | 15.38 | 1 | 7.69 | 0 | 0.00 | 3 | 23.08 | 1 | 7.69 |

|  | ***Do you believe resistance exercise can influence Inflammatory bowel disease in a positive way?*** | | | | | |
| --- | --- | --- | --- | --- | --- | --- |
|  | Yes | | No | | Don't know | |
| ***Disease activity*** | **N** | **%** | **N** | **%** | **N** | **%** |
| Remission | 38 | 39.18 | 4 | 4.12 | 55 | 56.70 |
| Mild | 43 | 38.74 | 3 | 2.70 | 65 | 58.56 |
| Moderate | 25 | 35.21 | 7 | 9.86 | 39 | 54.93 |
| Severe | 4 | 30.77 | 0 | 0.00 | 9 | 69.23 |

|  | ***Do you believe resistance exercise can influence Inflammatory bowel dis-ease in a negative way?*** | | | | | |
| --- | --- | --- | --- | --- | --- | --- |
|  | Yes | | No | | Don't know | |
| ***Disease activity*** | **N** | **%** | **N** | **%** | **N** | **%** |
| Remission | 8 | 8.25 | 32 | 32.99 | 57 | 58.76 |
| Mild | 8 | 7.21 | 30 | 27.03 | 73 | 65.77 |
| Moderate | 9 | 12.68 | 19 | 26.76 | 43 | 60.56 |
| Severe | 1 | 7.69 | 3 | 23.08 | 9 | 69.23 |

|  | ***Do you avoid certain types of exercise?*** | | | | | | | | | | | | | | | | | | | |
| --- | --- | --- | --- | --- | --- | --- | --- | --- | --- | --- | --- | --- | --- | --- | --- | --- | --- | --- | --- | --- |
|  | No | | Low intensity | | Moderate intensity | | Vigorous intensity | | Bodyweight exercise | | Structured weights classes | | Free weights | | Machine based weightlifting | | Other | | Combination | |
| ***Disease activity*** | **N** | **%** | **N** | **%** | **N** | **%** | **N** | **%** | **N** | **%** | **N** | **%** | **N** | **%** | **N** | **%** | **N** | **%** | **N** | **%** |
| Remission | 46 | 47.42 | 0 | 0.00 | 0 | 0.00 | 7 | 7.22 | 0 | 0.00 | 2 | 2.06 | 2 | 2.06 | 1 | 1.03 | 7 | 7.22 | 32 | 32.99 |
| Mild | 55 | 49.55 | 2 | 1.80 | 0 | 0.00 | 9 | 8.11 | 2 | 1.80 | 3 | 2.70 | 3 | 2.70 | 2 | 1.80 | 3 | 2.70 | 32 | 28.83 |
| Moderate | 26 | 36.62 | 2 | 2.82 | 2 | 2.82 | 14 | 19.72 | 0 | 0.00 | 0 | 0.00 | 0 | 0.00 | 0 | 0.00 | 3 | 4.23 | 24 | 33.80 |
| Severe | 5 | 38.46 | 0 | 0.00 | 0 | 0.00 | 1 | 7.69 | 0 | 0.00 | 1 | 7.69 | 0 | 0.00 | 0 | 0.00 | 3 | 23.08 | 3 | 23.08 |

|  | ***Would you consider undertaking whole body vibration exercise?*** | | | |
| --- | --- | --- | --- | --- |
|  | Yes | | No | |
| ***Disease activity*** | **N** | **%** | **N** | **%** |
| Remission | 129 | 91.49 | 12 | 8.51 |
| Mild | 158 | 93.49 | 11 | 6.51 |
| Moderate | 117 | 90.70 | 12 | 9.30 |
| Severe | 24 | 100.00 | 0 | 0.00 |
