# Supplementary material for: Perceptions, behaviours and barriers towards exercise practices in inflammatory bowel disease
Source: PLoS One. 2024 Apr 5;19(4):e0299228. doi: 10.1371/journal.pone.0299228 (PMC10997097; doi:10.1371/journal.pone.0299228)
Supplement: S3 Appendix — (DOCX) [file pone.0299228.s003.docx]

# Supplementary Materials 3

**Demographics/ Baseline**

**Q1. Please select your gender**

**- Female**

**- Male**

**- Non-binary**

**Q2. Please select your age**

**- 18-25**

**- 26-30**

**- 31-35**

**- 36-40**

**- 41-45**

**- 46-50**

**- 51-55**

**- 56-60**

**- 61-65**

**- 65+**

**Q3. Do you smoke?**

**- Yes**

**- No**

**Q4. Please select your current alcohol consumption (units per day): Note: 1 unit guideline = 220 ml cider, 76 ml wine/ 25 ml whisky/ 250 ml beer/ 275 ml alco pop.**

**- None**

**- 1 to 2**

**- 3 to 4**

**- 5 to 6**

**- 7 to 9**

**- 10+**

**- Other**

**Inflammatory bowel disease characteristics**

**Q5. Select the Inflammatory bowel disease modality that applies to you**

**- Ulcerative colitis**

**- Crohn’s Disease**

**- Other**

**Q6. How would you describe your current disease activity?**

**- Remission**

**- Mild**

**- Moderate**

**- Severe**

**Q7. Are you currently taking medication? (select more than one option if necessary)**

**- None**

**- Adalimumab**

**- Azathioprine and Mercaptopurine**

**- Biologic Drugs**

**- Infliximab**

**- Steroids**

**- Tofacitinib**

**- Ustekinumab**

**- Vedolizumab**

**- Aminosalicylates (5-ASAs)**

**- Other**

**Exercise perceptions, beliefs and behaviours**

**Q8. Do you exercise regularly?**

**- Yes**

**- No**

**Q9. Which type of aerobic exercise (i.e. exercises that increase your heart rate) do you mostly do?**

**- None**

**- Low intensity i.e. small increase in breathing rate, still easily able to hold a**

**- conversation**

**- Moderate intensity i.e. elevated breathing rate but still able to hold a conversation**

**- Vigorous intensity i.e. out of breath**

**Q10. Do you believe aerobic exercise can influence Inflammatory bowel disease in a positive way?**

**- Yes**

**- No**

**- Don’t know**

**Q11. Do you believe aerobic exercise can influence Inflammatory bowel disease in a negative way?**

**- Yes**

**- No**

**- Don’t know**

**Q12. Which type of resistance (e.g. muscle building, weightlifting and strength exercises) exercise do you usually do?**

**- None**

**- Body weight exercise e.g. Pilates, plank, press-up**

**- Structured weights classes e.g. studio weights**

**- Machine based weightlifting e.g. chest press, leg press**

**- Free weights e.g. back squat, bench press**

**- Other**

**Q13. Do you believe resistance exercise can influence Inflammatory bowel disease in a positive way?**

**- Yes**

**- No**

**- Don’t know**

**Q14. Do you believe resistance exercise can influence Inflammatory bowel disease in a negative way?**

**- Yes**

**- No**

**- Don’t know**

**Q15. Do you avoid certain kinds of exercise? (select more than one option if necessary)**

**- No**

**- Low intensity i.e. small increase in breathing rate, still easily able to hold a**

**- conversation**

**- Moderate intensity i.e. elevated breathing rate but still able to hold a conversation**

**- Vigorous intensity i.e. out of breath**

**- Body weight exercise e.g. Pilates, plank, press-up**

**- Structured weights classes e.g. studio weights**

**- Machine based weightlifting e.g. chest press, leg press**

**- Free weights e.g. back squat, bench press**

**- Other**

**Q16. What would/ does prevent you from engaging in exercise? (select more than one option if necessary)**

**- Fear of increased toilet urgency**

**- Fear of increased abdominal pain**

**- Fear of triggering a flare up**

**- Lack of scientific evidence**

**- Fatigue**

**- Pain during exercise**

**- Other**
