# Supplementary material for: Perceptions, behaviours and barriers towards exercise practices in inflammatory bowel disease
Source: PLoS One. 2024 Apr 5;19(4):e0299228. doi: 10.1371/journal.pone.0299228 (PMC10997097; doi:10.1371/journal.pone.0299228)
Supplement: Attachment — Submitted filename: PLOS_ONE_SUBMIT.docx [file pone.0299228.s004.docx]

# **Perceptions, behaviours and barriers towards exercise practices in inflammatory bowel disease**

Jonathan Sinclair^a^, Johanne Brooks-Warburton^b,c^ & Lindsay Bottoms^d^

a. Research Centre for Applied Sport, Physical Activity and Performance, School of Sport & Health Sciences, Faculty of Allied Health and Wellbeing, University of Central Lancashire, Lancashire, PR1 2HE, UK.
b. Gastroenterology Department, Lister Hospital, Stevenage, Hertfordshire, UK.
c. School of Life and Medical Sciences, University of Hertfordshire, Hertfordshire, AL10 9AB, UK.
d. Centre for Research in Psychology and Sport Sciences, School of Life and Medical Sciences, University of Hertfordshire, Hertfordshire, AL10 9AB, UK.

**Corresponding author: Dr Lindsay Bottoms** [**l.bottoms@herts.ac.uk**](mailto:l.bottoms@herts.ac.uk) **@BottomsLindsay**

**ORCID:**

Dr Jonathan Sinclair <https://orcid.org/0000-0002-2231-3732>

Dr Johanne Brooks-Warburton	<http://orcid.org/0000-0003-1705-6476>

Dr Lindsay Bottoms <https://orcid.org/0000-0003-4632-3764>

# **Abstract**

Inflammatory bowel disease (IBD), a chronic disease affecting the digestive tract, has a significant impact on health-related quality of life. Pharmaceutical treatment is typically adopted, yet exercise is increasingly becoming recognized as an adjunct therapy. This study aimed to explore the perspectives, behaviours, and barriers of IBD patients in terms of their exercise habits. A 16-item closed-ended questionnaire was completed by 463 adult IBD patients (Ulcerative colitis = 57.02%, Crohn’s dis-ease = 40.60% and Other = 2.38%) (Female = 76.67%, Male = 22.46 and Non-binary = 0.86%). The questionnaire was divided into three sections: baseline/demographic characteristics, disease characteristics, and exercise perceptions, beliefs, and behaviours. Significantly (P<0.001) more participants (63.07%) reported that they engage regularly with exercise compared to those who do not; however, engagement was significantly lower in female patients (59.72%) compared to males (74.04%). Respondents also rated significantly (P<0.001) that a combination of factors prevents engagement in exercise (74.30%). Moderate intensity exercise was the predominant (P<0.001) aerobic modality (39.04%), the majority (P<0.001) response was that patients undertake no resistance training (27.74%), and significantly more (P<0.001) patients indicated that they don’t know whether resistance training can influence IBD either positively (57.53%) or negatively (62.33%). Whilst it is encouraging that IBD patients are engaging regularly with exercise, the reduced levels of engagement in females and lack of knowledge/ engagement with resistance training, indicate that future implementation and educational developments are necessary to enhance exercise in females and resistance training engagement in all IBD patients.

**Keywords:** Inflammatory bowel disease; Ulcerative colitis; Crohn’s disease; Exercise.

# **Introduction**

Inflammatory bowel diseases (IBD) are a group of chronic diseases that affect the digestive tract [1]. The two most common types of IBD are ulcerative colitis (UC) and Crohn's disease (CD) [2]. UC is characterized by inflammation of the lining of the colon, whilst CD can cause inflammation anywhere along the digestive tract [2]. The primary symptoms associated with IBD are fatigue, pain and diarrhoea [3]. Inflammation of the gut wall can also lead to malabsorption, which can cause low bone mineral density [4] and loss of muscle mass [5]. Other extraintestinal manifestations can develop such as osteoarthritis uveitis, iritis, episcleritis, erythema nodosum and pyroderma gangernosum [3,6].

The incidence of IBD is increasing worldwide [7], and the annual monetary impact of the disease in the United States alone is over $31 billion [8]. IBD can have a significant impact on a person's quality of life, including their psychological wellbeing, body image, social interactions, relationships with friends and family [9]. IBD can also lead to diminished labour force participation; further increasing the fiscal burden of the disease [10]. The clinical spectrum of IBD is wide and ranges from an asymptomatic quiescent state to life-threatening severe illness [1].

Clinical management of IBD focuses on inducing and maintaining remission, im-proving quality of life and prevention of complications and the necessity for surgery [11]. At present, pharmaceutical intervention is typically adopted as the primary approach to disease management [12]. Traditionally, the pharmaceutical armamentarium has included immunosuppressants such as Azathioprine and Mercaptopurine, Biologic drugs such as Infliximab, Tofacitinib, Ustekinumab, Vedolizumab and Adalimumab, Steroids, and Oral 5-aminosalicylates [11, 13]. Furthermore, although, significant progress has been made in the pharmaceutical management of IBD, surgical intervention remains an important means for IBD treatment [13]. A recent investigation has shown that the rate of surgery for CD is 8.8% and that for UC is 7.5% [14].

The pathogenesis of IBD is multifactorial in nature, with complex interaction between genetics, environmental and lifestyle factors, and the immune system [10]. Importantly geographical patterns of IBD epidemiology show the highest prevalence and acceleration in developed nations [15], intimating that Westernized lifestyles appear to be connected to IBD aetiology [16, 17]. Western lifestyles have increasingly been characterized by a lack of regular exercise [18], and lack of exercise has notably been high-lighted as a risk factor for the development of IBD [19]. Importantly, regular exercise is being advocated as an adjunct therapy in IBD [20]. Intervention studies indicate that low-moderate intensity aerobic exercise is efficacious in IBD patients by increasing health-related quality of life, reducing inflammation, reducing fatigue, increasing bone mineral density and improving psychological wellbeing [21-25], without significant adverse effects [26]. Although the exact biological mechanism remains unidentified, alteration in gut microbiota and the influence of exercise on immunological and inflammatory processes have been offered as potential routes of action [27].

Current guidelines in relation to exercise in adults with IBD, advocate low-moderate levels of exercise of 20–60 minutes in duration on 2–5 days per week [28]. However, these recommendations were introduced at a time when research exploring the efficacy of exercise in IBD was in its infancy and have a theoretical underpinning developed based on benefits of exercise observed in healthy individuals [29]. As such current IBD exercise guidelines do not take into account the therapeutic benefits of potentially important contemporary modalities such as high-intensity exercise [20, 30] or resistance training [31, 32].

Notwithstanding the favourable influence of exercise in IBD; patients have been shown to undertake significantly reduced levels of exercise following diagnosis [33]. A limited number of analyses have attempted to quantify both physical activity and exercise behaviours and barriers in IBD patient populations, although there is considerable variation between studies. Two separate cross-sectional analyses conducted in Italy have shown that 20.6% [34] and 25.6% [35] of patients regularly engaged in physical activity. Furthermore, in Canada, the findings from a population-based investigation showed that in Crohn’s patients 58.0%, 24.1% and 17.9% and in UC patients 53.6%, 25.4% and 21.0% identified as inactive, moderately active and active respectively [36]. A survey undertaken in UK by the Crohn’s and Colitis Foundation showed that 66% of patients participated in exercise, with 32% reporting exercising daily, 57% weekly, 5% monthly, and 5% less than monthly [37]. In a study undertaken in the United States, 16.4% of patients never exercised, 32.8% exercised 1–2 times per week, 23.6% 3–4 times per week, and 18.0% exercised >4 times per week [38]. Finally, a recent investigation (which did not specify the location of its patients), showed that 17% of patients were highly physically active, 50% were minimally active, and 33% were inactive [29]. Whilst previous analyses have quantified physical activity and exercise behaviours, there has been comparatively little consideration of barriers and perceptions towards exercise in IBD patients. Further research is required in order to better understand exercise perceptions, behaviours and barriers in IBD patients. Specifically, exploring the extent to which these important factors differ among bespoke patient subgroups (i.e. UC and CD), disease activity and gender, as well as towards distinct training modalities such as aerobic exercise, resistance training and high-intensity exercise, will assist in the design/tailoring of interventions to improve long-term remission maintenance and quality of life in IBD patients.

Pharmaceutical intervention is the foremost approach to IBD management [12]. However, medicinal therapies for IBD typically must be taken continuously [27], are accompanied by substantial side-effects [39] and notably associated with low levels of adherence [40]. Additional approaches to treatment in IBD are increasingly being sought [41], and as many as 56% of patients have been shown to seek alternative or complimentary treatment modalities [42]. Being able to prescribe a specific exercise regimen for IBD patients as an adjunct therapy could improve patient’s health-related quality of life at a lower economic cost. Improved understanding of the perceptions, beliefs and barriers to exercise, could assist in the implementation of new exercise therapies in this patient population. Therefore, this investigation aims to gain new insight into perceptions, behaviours and barriers in IBD patient's regarding their exercise practices.

# **Materials and Methods**

## **Ethical considerations**

The study was approved by the Institutional ethical board of the University of Central Lancashire reference: HEALTH 0423. All participants provided informed consent in accordance with the Declaration of Helsinki.

## **Study design and participants**

This study used an observational and quantitative approach to achieve its objectives. IBD patients anonymously completed a self-administered online questionnaire hosted on the Jisc platform (Jisc, UK). Recruitment was undertaken in the in the United Kingdom through Twitter and Facebook based social media platforms, using general and research specific accounts belonging to Crohn’s and Colitis UK. Participants were eligible to participate in the study if they were over 18 years of age and had been diagnosed with Crohn's disease (CD), ulcerative colitis (UC), or another IBD condition. The survey was available online for 3 months, from March 1st 2023 to June 1st 2023. A total of 463 responses were received (UC = 264, CD = 188, Other = 11) after the survey period ended.

## **Questionnaire**

The questionnaire was created after reviewing scientific studies on exercise in IBD patients. This facilitated the identification of relevant topics and development of questions and response options. In accordance with our previous questionnaire-based analyses, four research and clinical experts in the field of IBD reviewed the survey and provided feedback to improve its face validity [41, 43]. Two of the aforementioned experts and one of the authors of the paper importantly have lived experience of IBD. This feedback was incorporated into the questionnaire design before it was submitted for ethical approval. The options for respondents in relation to medication questions were developed based on the current guidelines of Chron's & Colitis UK and the British Society of Gastroenterology consensus guidelines, in consultation with the aforementioned clinical professionals from within this field [44].

The questionnaire, excluding the information page and consent options, featured 16 close-ended questions divided into three sub-sections. Section 1 related to participants' baseline demographics, such as gender, age, smoking, and alcohol use. Section 2 explored disease characteristics, such as the specific IBD condition, disease activity, and current medication. Section 3 considered exercise perceptions, beliefs, and behaviours.

The section relating to exercise perceptions, beliefs, and behaviours included a series of questions arranged as following in chronological order; whether patients exercise regularly, the specific type of aerobic exercise they engaged in, their perceptions regarding potential positive or negative impact of aerobic exercise on IBD, the type of resistance training they performed, and their perceptions regarding potential effects of resistance training on IBD. Additionally, the questionnaire asked about the avoidance of certain types of exercise. Patients who reported that they do not engage in regular exercise regularly were directed to the question about avoiding certain kinds of exercise. The questionnaire is presented in Supplementary materials 3.

## **Statistical analyses**

The data from the questionnaire were inputted into SPSS v27 (IBM), and categorical data for each survey question were coded. When reporting proportions for categorical variables, both the total count (N) and the corresponding percentages (%) were provided. One-sample chi-square (X^2^) goodness of fit tests were employed for all questions in the exercise perceptions, beliefs, and behaviours section, as well as the questions related to smoking behaviour, alcohol consumption, and current medication [41]. These tests were used to compare the proportions of participants who selected each response option. In addition, two-way Pearson chi-square tests of independence were used to perform bivariate comparisons. Specifically, these tests were conducted to assess differences in responses to each question based on baseline/demographic variables, gender, age, specific IBD condition and disease activity [41]. For the sake of brevity, only the two-way Pearson chi-square tests of independence that yielded statistically significant results were presented in the results. Statistical significance for all analyses was set at the P<0.05 level.

# **Results**

## **Baseline/demographic characteristics**

The baseline/demographic characteristics of the study population are described in Table 1. Overall, there were 463 eligible patients in the survey, of whom 76.67% were female, 22.46% male and 0.86% non-binary. In addition, 57.02% of patients had UC, 40.60% CD and 2.38% another IBD condition. Overall, 30.45% of patients were in remission, 36.50% experienced mild, 27.86%, moderate and 5.18% severe symptoms.

Table 1. Baseline/demographic characteristics.

| **Variables** | **N** | **%** |
| --- | --- | --- |
| ***Gender*** |  |  |
| Female | 355 | 76.67 |
| Male | 104 | 22.46 |
| Non-binary | 4 | 0.86 |
| ***Age*** |  |  |
| 18-25 | 45 | 9.72 |
| 26-30 | 44 | 9.50 |
| 31-35 | 59 | 12.74 |
| 36-40 | 84 | 18.14 |
| 41-45 | 65 | 14.04 |
| 46-50 | 66 | 14.25 |
| 51-55 | 45 | 9.72 |
| 56-60 | 24 | 5.18 |
| 61-65 | 22 | 4.75 |
| 65+ | 9 | 1.94 |
| ***Disease*** |  |  |
| UC | 264 | 57.02 |
| CD | 188 | 40.60 |
| Other | 11 | 2.38 |
| ***Disease activity*** |  |  |
| Remission | 141 | 30.45 |
| Mild | 169 | 36.50 |
| Moderate | 129 | 27.86 |
| Severe | 24 | 5.18 |

## **Disease characteristics**

The disease characteristics of the study population are described in Table 2. One-sample chi-square goodness of fit tests showed that there were significant differences in responses for ‘Medication’ (*X*^2^ _(11)_ = 440.16, P<0.001) with significantly more participants taking a combination of medications. Finally, there were also significant differences in responses for both ‘Smoking behaviour’ (*X*^2^ _(1)_ = 316.82, P<0.001) with the majority of participants being non-smokers and also ‘Alcohol’ consumption (*X*^2^ _(6)_ = 955.28, P<0.001) which showed that the majority of participants consumed no alcohol. No further statistically significant findings were observed.

Table 2. Disease characteristics (* = Significant chi-squared test).

| **Variables** | **N** | **%** |  |
| --- | --- | --- | --- |
| ***Medication*** |  |  |  |
| None | 59 | 12.74 | * |
| Adalimumab | 36 | 7.78 |  |
| Azathioprine and Mercaptopurine | 35 | 7.56 |  |
| Biologic Drugs | 5 | 1.08 |  |
| Infliximab | 13 | 2.81 |  |
| Steroids | 39 | 8.42 |  |
| Tofacitinib | 1 | 0.22 |  |
| Ustekinumab | 26 | 5.62 |  |
| Vedolizumab | 19 | 4.10 |  |
| Aminosalicylates (5-ASAs) | 26 | 5.62 |  |
| Other | 54 | 11.66 |  |
| Combination | 150 | 32.4 |  |
| ***Smoke*** |  |  | * |
| Yes | 40 | 8.64 |  |
| No | 423 | 91.36 |  |
| ***Alcohol (units/week)*** |  |  |  |
| None | 281 | 60.69 |  |
| 1-2 | 118 | 25.49 |  |
| 3-4 | 20 | 4.32 |  |
| 5-6 | 15 | 3.24 | * |
| 7-9 | 9 | 1.94 |  |
| 10+ | 7 | 1.51 |  |
| Other | 13 | 2.81 |  |

## **Exercise perceptions, beliefs and behaviours (one-sample analyses)**

The exercise perceptions, beliefs and barriers of the study population are described in Tables 3-4. There were significant differences in responses for ‘Do you exercise regularly’ (*X*^2^ _(1)_ = 31.62, P<0.001) which showed that significantly more participants are regularly engaged with exercise activities and also ‘What would/ does prevent you from engaging in exercise’ (*X*^2^ _(7)_ = 1673.17, P<0.001) which showed that significantly more participants indicated that a combination of factors prevent engagement in exercise. Of those who exercise regularly there was a significant difference in responses for ‘Which type of aerobic exercise do you mostly do’ (*X*^2^ _(3)_ = 84.66, P<0.001) which revealed that significantly more participants engaged in moderate intensity exercise.

In addition, there were significant differences in responses for ‘Do you believe aerobic exercise can influence Inflammatory bowel disease in a positive way‘ (*X*^2^ _(2)_ = 126.30, P<0.001) which showed that significantly more participants felt that aerobic exercise has a positive influence on Inflammatory bowel disease and for ‘Do you believe aerobic exercise can influence Inflammatory bowel disease in a negative way’ (*X*^2^ _(2)_ = 7.24, P=0.027) which revealed that significantly more participants indicated that they do not know if aerobic exercise can negatively influence Inflammatory bowel disease.

There was a significant difference in responses for ‘Which type of resistance exercise do you mostly do’ (*X*^2^ _(5)_ = 68.49, P<0.001) which revealed that significantly more participants engaged in no resistance training. In addition, there were significant differences in responses for ‘Do you believe resistance exercise can influence Inflammatory bowel disease in a positive way‘ (*X*^2^ _(2)_ = 124.30, P<0.001) and ‘Do you believe resistance exercise can influence Inflammatory bowel disease in a negative way’ (*X*^2^ _(2)_ = 127.75, P<0.001) which revealed that significantly more participants indicated that they do not know if resistance exercise can positively or negatively influence Inflammatory bowel disease.

There was a significant difference in responses for ‘Do you avoid certain types of exercise’ (*X*^2^ _(9)_ = 633.21, P<0.001), which revealed that significantly more participants did not avoid any exercise modality. No further statistically significant findings were observed.

Table 3. Exercise perceptions, behaviours and barriers (* = Significant chi-squared test).

| **Variables** | **N** | **%** |  |
| --- | --- | --- | --- |
| ***Do you exercise regularly?*** |  |  | * |
| Yes | 292 | 63.07 |  |
| No | 171 | 36.93 |  |
| ***What would/ does prevent you from engaging in exercise?*** |  |  | * |
| Combination of factors | 344 | 74.30 |  |
| Fatigue | 64 | 13.82 |  |
| Fear of increased toilet urgency | 28 | 6.05 |  |
| Other | 20 | 4.32 |  |
| Pain during exercise | 3 | 0.65 |  |
| Fear of triggering a flare up | 2 | 0.43 |  |
| Lack of scientific evidence | 1 | 0.22 |  |
| Fear of increased abdominal pain | 1 | 0.22 |  |
| ***Which type of aerobic exercise to you mostly do?*** |  |  | * |
| Moderate intensity | 114 | 39.04 |  |
| Low intensity | 96 | 32.88 |  |
| Vigorous | 72 | 24.66 |  |
| None | 10 | 3.42 |  |
| ***Do you believe aerobic exercise can influence Inflammatory bowel disease in a positive way?*** |  |  | * |
| Yes | 168 | 57.53 |  |
| Don’t know | 111 | 38.01 |  |
| No | 13 | 4.45 |  |
| ***Do you believe aerobic exercise can influence Inflammatory bowel disease in a negative way?*** |  |  | * |
| Don’t know | 119 | 40.75 |  |
| Yes | 87 | 29.79 |  |
| No | 86 | 29.45 |  |
| ***Which type of resistance exercise to you mostly do?*** |  |  | * |
| None | 81 | 27.74 |  |
| Bodyweight | 73 | 25.00 |  |
| Free weights | 59 | 20.21 |  |
| Machines | 34 | 11.64 |  |
| Other | 24 | 8.22 |  |
| Structured classes | 21 | 7.19 |  |

## **Exercise perceptions, beliefs and behaviours (two-way cross-tabulation analyses)**

The exercise perceptions, beliefs and behaviours of the study population as a function of the baseline/demographic groups that exhibited statistical significance when examined using two-way cross-tabulation analyses are described in Supplementary materials 1A-E. Those that did not exhibit statistical significance are presented collectively in Supplementary materials 2. These tables are presented as appendices owing to their size.

A significant difference (*X*^2^ _(2)_ = 8.17, P=0.004) in the responses to ‘Do you exercise regularly’ as a function of ‘Gender’ was observed, with a greater proportion of males indicating that they engage with exercise on a regular basis. There was also a significant difference (*X*^2^ _(10)_ = 26.72, P=0.003) in the responses to ‘Which type of resistance exercise to you mostly do’ as a function of ‘Gender’ was observed, with a greater proportion of males utilizing machine-based resistance training and correspondingly a greater proportion of females attending structured resistance training classes.

There was also a significant difference (*X*^2^ _(9)_ = 25.13, P=0.003) in the responses to ‘Which type of aerobic exercise to you mostly do’ as a function of ‘Disease activity’, with a greater proportion of those with severe symptoms engaging in no aerobic exercise. There was also a significant difference (*X*^2^ _(9)_ = 34.52, P=0.032) in the responses to ‘What would/ does prevent you from engaging in exercise’ as a function of ‘Disease activity’, with a greater proportion of those with severe symptoms indicating that fear of increased toilet urgency prevents engagement in exercise and those in remission denoting that other factors were responsible. No further statistically significant findings were observed.

Table 4. Exercise perceptions, behaviours and barriers (* = Significant chi-squared test).

| **Variables** | **N** | **%** |  |
| --- | --- | --- | --- |
| ***Do you believe resistance exercise can influence Inflammatory bowel disease in a positive way?*** |  |  | * |
| Don’t know | 168 | 57.53 |  |
| Yes | 110 | 37.67 |  |
| No | 14 | 4.79 |  |
| ***Do you believe resistance exercise can influence Inflammatory bowel disease in a negative way?*** |  |  | * |
| Don’t know | 182 | 62.33 |  |
| No | 84 | 28.77 |  |
| Yes | 26 | 8.90 |  |
| ***Do you avoid certain types of exercise?*** |  |  | * |
| No | 132 | 45.21 |  |
| Combination | 91 | 31.16 |  |
| Vigorous intensity | 31 | 10.62 |  |
| Other | 16 | 5.48 |  |
| Structured weights classes | 6 | 2.05 |  |
| Free weights | 5 | 1.71 |  |
| Low intensity | 4 | 1.37 |  |
| Machine based weightlifting | 3 | 1.03 |  |
| Moderate intensity | 2 | 0.68 |  |

# **Discussion**

The aim of the current investigation was to explore the perceptions, behaviours and barriers of IBD patients in relation to their exercise practices. Furthermore, this study also sought to examine IBD patients’ perceptions regarding their willingness to engage with WBV therapy. This investigation provides information that may be important for the implementation of future initiatives seeking to improve disease management and quality of life.

Importantly, the current investigation showed that significantly more participants are regularly engaged with exercise activities with 63.07%% of total IBD patients indicating that they take part in exercise. This is a notable observation, as previous randomized trials and correlational analyses have shown that exercise is associated with improvements in health-related quality of life in IBD patients [19, 20]. The exercise engagement levels observed in the current study are noticeably greater than those observed in earlier (i.e. prior to 2011) investigations exploring physical activity [34-36] before the body of publications concerning intervention research highlighting the efficacy of exercise in IBD [29]. However, the exercise engagement observations from this investigation are in agreement with more recent (i.e. post 2014) examinations both physical activity and exercise specific behaviours [29, 37, 38], indicating positively that the findings from randomized intervention trials and patient education information do appear to now be translating into practice.

However, (notwithstanding the distinctions between physical activity and exercise) the findings from this investigation importantly show that in relation to the general public, IBD patients appear to engage less frequently as the recent Sport England active lives survey showed that 74.2% of adults are either fairly (30-149 minutes/week) or very active (>150 minutes/week) [45]. The analyses of the barriers to physical activity indicated immoderately that a combination of factors prevent them from engaging in exercise, indicating that a multifactorial approach to improving exercise activity is necessary. Furthermore, whilst the two-way cross-tabulation analyses showed no differences in exercise engagement between disease type, disease activity or age; female patients were significantly less likely to exercise than males, with over 40% not engaging in exercise. This is an important observation, particularly as further inspection of the cross-tabulation analyses of the question in relation to barriers to exercise did not reveal any statistical differences as a function of gender. Regardless, taking into account the positive influence of exercise on health related and indeed other quality of life indices, it is important for future implementation initiatives to be undertaken in order to enhance exercise engagement in female IBD patients.

In relation to exercise modality, this investigation indicated that moderate level exercise was the predominant aerobic modality, but the principal response in relation to resistance training was that IBD patients do not engage with this form of exercise. Furthermore, the cross-tabulation analyses showed that males were more likely to utilize machine-based resistance training whereas females were most likely to undertake structured resistance training classes. Whilst it remains unknown as to which is the most suitable resistance training modality for IBD, it is important to recognise that patients’ responses also highlighted a statistical lack of knowledge in relation to the positive and negative effective effects of resistance training in IBD. These observations support the responses to the type of resistance exercise that patients engaged with. This is a prominent observation, as resistance training has been shown to enhance both bone mineral density and skeletal muscle mass in IBD, which are renowned symptoms in this patient group [31, 32]. It should be acknowledged that in relation to aerobic exercise, intervention analyses examining the effects of resistance training is far less established. Therefore, it is important that further randomized intervention trials are conducted exploring the effects of resistance training on pertinent IBD indices alongside additional educational developments to translate these observations into patients own habitual disease management activities.

Further exploration of the two-way cross-tabulation analyses showed that although no differences were observed as a function of disease type, a significantly greater proportion of those with severe symptoms engage in no aerobic exercise. This observation is perhaps to be expected as intuitively it could be anticipated that those experiencing extremely pronounced disease activity would be less able/ willing to participate in exercise. Examination of the mechanisms involved in prevention from engagement in exercise appear to provide some clarification for this finding, as a significantly greater proportion of those with severe symptoms indicated that fear of increased toilet urgency was responsible for their lack of exercise engagement. This is important to note, when clinicians or exercise professionals are recommending exercise to adults with IBD. They should understand whether toilet urgency is a barrier for the patient. If toilet urgency is an issue, then recommending exercise with a known toilet location such as home-based exercise or some form of indoor exercise such as the gym could be preferential where the patient knows how long it will take to get to the toilet when starting the exercise.

As with any study, this investigation is not without limitations, which should be acknowledged. Whilst part of the aims of this investigation were to examine patients’ perceptions, the findings in relation to behaviours and other important parameters such as disease activity could have been affected due to the self-reported nature of data. As IBD patients in this investigation were recruited exclusively through UK based social media advertisements, this means that like all online surveys, it is not possible to eliminate selection bias, and the results may not be directly generalizable to a wider IBD population. Of particular note, is that 76.67%% of survey patients were female, meaning that although exercise parameters as a function of gender were considered as part of the two-way chi-squared analyses, males and in particular non-binary patients were underrepresented in this investigation.

# **Conclusion**

In conclusion, the current investigation showed that significantly more participants engaged with exercise than did not, although not at the level undertaken by the general public in the UK. Examination of the barriers associated with exercise engagement revealed that a combination of factors prevents engagement in exercise, indicating that a multifactorial approach to improving exercise activity is necessary. Importantly, this study showed that females were significantly less likely to exercise than males, suggesting that future implementation initiatives be undertaken to enhance exercise participation in female IBD patients. In addition, patients with severe symptoms predominantly engaged in no aerobic exercise and denoted that fear of increased toilet urgency was responsible for their lack of exercise engagement. Therefore, it appears that to allow the benefits of exercise to be manifested, finding exercise modalities which have easy access to a toilet is required to help alleviate the stress of toilet urgency. In relation to exercise modality, this investigation indicated that moderate level exercise was the predominant aerobic modality, but the principal response in relation to resistance training was that IBD patients do not engage with this form of exercise, owing potentially to a lack of under-standing regarding the positive/ negative effects of this training modality. Given the previously observed benefits of resistance training in IBD, additional educational developments are necessary to translate this activity into patients exercise based disease management activities.

# **Acknowledgments**

We would like to thank Crohn’s and Colitis UK for distributing the survey.

# **References**

1. Chan D, Robbins H, Rogers S, Clark S, Poullis A. Inflammatory bowel disease and exercise: results of a Crohn's and Colitis UK survey. Frontline Gastroenterology. 2014;5(1):44-48.
2. Fakhoury M, Negrulj R, Mooranian A, Al-Salami H. Inflammatory bowel disease: clinical aspects and treatments. J Inflamm Res. 2014;7:113-120.
3. Bilski J, Brzozowski B, Mazur-Bialy A, Sliwowski Z, Brzozowski T. The role of physical exercise in inflammatory bowel disease. BioMed Res Int. 2014;2014:429031. PMID: 24877092.
4. Lima CA, Lyra AC, Mendes CMC, Lopes MB, Coqueiro FG, Rocha R, Santana GO. Bone mineral density and inflammatory bowel disease severity. Braz J Med Biol Res. 2017;50(12):e6374.
5. Ryan E, McNicholas D, Creavin B, Kelly ME, Walsh T, Beddy D. Sarcopenia and inflammatory bowel disease: a systematic review. Inflamm Bowel Dis. 2019;25(1):67-73.
6. Gajendran M, Loganathan P, Catinella AP, Hashash JG. A comprehensive review and update on Crohn's disease. Dis Mon. 2018;64(2):20-57.
7. Burisch J, Munkholm P. Inflammatory bowel disease epidemiology. Curr Opin Gastroenterol. 2013;29(4):357-362.
8. Mehta F. Report: economic implications of inflammatory bowel disease and its management. Am J Manag Care. 2016;22(3):51-60.
9. Su S, Marrie RA, Bernstein CN. Factors associated with social participation in persons living with inflammatory bowel disease. J Can Assoc Gastroenterol. 2022;5(2):59-67.
10. Bianco AM, Girardelli M, Tommasini A. Genetics of inflammatory bowel disease from multifactorial to monogenic forms. World J Gastroenterol. 2015;21(43):12296.
11. Speight RA, Mansfield JC. Drug advances in inflammatory bowel disease. Clin Med (Lond). 2013;13(4):378-382.
12. Chumanevich AA, Chaparala A, Witalison EE, Tashkandi H, Hofseth AB, Lane C, Chumanevich AA. Looking for the best anti-colitis medicine: A comparative analysis of current and prospective compounds. Oncotarget. 2017;8(1):228-237.
13. Cai Z, Wang S, Li J. Treatment of inflammatory bowel disease: a comprehensive review. Front Med. 2021;8:765474.
14. Lowe SC, Sauk JS, Limketkai BN, Kwaan MR. Declining rates of surgery for inflammatory bowel disease in the era of biologic therapy. J Gastrointest Surg. 2021;25:211-219.
15. Marion-Letellier R, Savoye G, Ghosh S. IBD: in food we trust. J Crohns Colitis. 2016;10(11):1351-1361.
16. Rizzello F, Spisni E, Giovanardi E, Imbesi V, Salice M, Alvisi P, Gionchetti P. Implications of the Westernized Diet in the Onset and Progression of IBD. Nutrients. 2019;11(5):1033.
17. Lamers CR, De Roos NM, Heerink HH, Van de Worp-Kalter LA, Witteman BJ. Lower impact of disease on daily life and less fatigue in patients with inflammatory bowel disease following a lifestyle intervention. Inflamm Bowel Dis. 2022;28(12):1791-1799.
18. Booth FW, Hawley JA. The erosion of physical activity in Western societies: an economic death march. Diabetologia. 2015;58:1730-1734.
19. Basil M, Guo L, Groshek J, Reich J, Farraye FA. Poor Knowledge and High Stigma of IBD in the General Population: Results of a National Survey. Off J Am Coll Gastroenterol ACG. 2016;111:335.
20. Tew GA, Leighton D, Carpenter R, Anderson S, Langmead L, Ramage J, Bottoms L. High-intensity interval training and moderate-intensity continuous training in adults with Crohn’s disease: a pilot randomised controlled trial. BMC Gastroenterol. 2019;19(1):1-11.
21. D'inca R, Varnier M, Mestriner C, Martines D, D'Odorico A, Sturniolo GC. Effect of moderate exercise on Crohn's disease patients in remission. Ital J Gastroenterol Hepatol. 1999;31(3):205-210.
22. Loudon CP, Corroll V, Butcher J, Rawsthorne P, Bernstein CN. The effects of physical exercise on patients with Crohn’s disease. Am J Gastroenterol. 1999;94(3):697-703.
23. Ng V, Millard W, Lebrun C, Howard J. Low-intensity exercise improves quality of life in patients with Crohn's disease. Clin J Sport Med. 2007;17(5):384-388.
24. Klare P, Nigg J, Nold J, Haller B, Krug AB, Mair S, Huber W. The impact of a ten-week physical exercise program on health-related quality of life in patients with inflammatory bowel disease: a prospective randomized controlled trial. Digestion. 2015;91(3):239-247.
25. Robinson RJ, Krzywicki T, Almond L, Al–Azzawi F, Abrams K, Iqbal SJ, Mayberry JF. Effect of a low-impact exercise program on bone mineral density in Crohn's disease: a randomized controlled trial. Gastroenterology. 1998;115(1):36.
26. Bottoms L, Leighton D, Carpenter R, Anderson S, Langmead L, Ramage J, Tew G. Affective and enjoyment responses to 12 weeks of high-intensity interval training and moderate continuous training in adults with Crohn’s disease. PLoS One. 2019;14(9):e0222060.
27. Engels M, Cross RK, Long MD. Exercise in patients with inflammatory bowel diseases: current perspectives. Clin Exp Gastroenterol. 2017;11:1-11.
28. Ball E. Exercise guidelines for patients with inflammatory bowel disease. Gastroenterol Nurs. 1998;21(3):108-111.
29. Tew GA, Jones K, Mikocka-Walus A. Physical activity habits, limitations, and predictors in people with inflammatory bowel disease: a large cross-sectional online survey. Inflamm Bowel Dis. 2016;22(12):2933-2942.
30. Ploeger H, Obeid J, Nguyen T, Takken T, Issenman R, de Greef M, Timmons B. Exercise and inflammation in pediatric Crohn’s disease. Int J Sports Med. 2012;33(08):671-679.
31. de Souza Tajiri GJ, de Castro CLN, Zaltman C. Progressive resistance training improves muscle strength in women with inflammatory bowel disease and quadriceps weakness. J Crohn's Colitis. 2014;8(12):1749-1750.
32. Jones K, Baker K, Speight RA, Thompson NP, Tew GA. Randomised clinical trial: combined impact and resistance training in adults with stable Crohn's disease. Aliment Pharmacol Ther. 2020;52(6):964-975.
33. Gatt K, Schembri J, Katsanos KH, Christodoulou D, Karmiris K, Kopylov U, Ellul P. Inflammatory bowel disease [IBD] and physical activity: a study on the impact of diagnosis on the level of exercise amongst patients with IBD. J Crohn's Colitis. 2019;13(6):686-692.
34. D’Inca R, Garribba AT, Vettorato MG, Martin A, Martines D, Di Leo V, Sturniolo GC. Use of alternative and complementary therapies by inflammatory bowel disease patients in an Italian tertiary referral centre. Dig Liver Dis. 2007;39(6):524-529.
35. Bertomoro P, Renna S, Cottone M, Riegler G, Bossa F, Giglio L, D'Incà R. Regional variations in the use of complementary and alternative medicines (CAM) for inflammatory bowel disease patients in Italy: An IG-IBD study. J Crohn's Colitis. 2010;4(3):291-300.
36. Mack DE, Wilson PM, Gilmore JC, Gunnell KE. Leisure-time physical activity in Canadians living with Crohn disease and ulcerative colitis: population-based estimates. Gastroenterol Nurs. 2011;34(4):288-294.
37. Chan D, Robbins H, Rogers S, Clark S, Poullis A. Inflammatory bowel disease and exercise: results of a Crohn's and Colitis UK survey. Frontline Gastroenterology. 2014;5(1):44-48.
38. DeFilippis EM, Tabani S, Warren RU, Christos PJ, Bosworth BP, Scherl EJ. Exercise and self-reported limitations in patients with inflammatory bowel disease. Dig Dis Sci. 2016;61:215-220.
39. Atreya R, Neurath MF, Siegmund B. Personalizing treatment in IBD: hype or reality in 2020? Can we predict response to anti-TNF? Front Med. 2020;7:517.
40. Kane SV, Cohen RD, Aikens JE, Hanauer SB. Prevalence of nonadherence with maintenance mesalamine in quiescent ulcerative colitis. Am J Gastroenterol. 2001;96(10):2929-2933.
41. Sinclair J, Dillon S, Bottoms L. Perceptions, beliefs and behaviors of nutritional and supplementary practices in inflammatory bowel disease. Sport Sci Health. 2022;18(4):1301-1310.
42. Weizman AV, Ahn E, Thanabalan R, Leung W, Croitoru K, Silverberg MS, Nguyen GC. Characterisation of complementary and alternative medicine use and its impact on medication adherence in inflammatory bowel disease. Aliment Pharmacol Ther. 2012;35(3):342-349.
43. Allan R, Akin B, Sinclair J, Hurst H, Alexander J, Malone JJ, Ihsan M. Athlete, coach and practitioner knowledge and perceptions of post-exercise cold-water immersion for recovery: a qualitative and quantitative exploration. Sport Sci Health. 2021;18:699–713.
44. Lamb CA, Kennedy NA, Raine T, Hendy PA, Smith PJ, Limdi JK, IBD Guidelines eDelphi Consensus Group. British Society of Gastroenterology consensus guidelines on the management of inflammatory bowel disease in adults. Gut. 2019;68(3):1-106.
45. Sport England. Active Lives Adult Survey November 2020–21 Report. Sport England: London, UK. 202
