# Supplementary material for: Perceptions, behaviours and barriers towards exercise practices in inflammatory bowel disease
Source: PLoS One. 2024 Apr 5;19(4):e0299228. doi: 10.1371/journal.pone.0299228 (PMC10997097; doi:10.1371/journal.pone.0299228)
Supplement: Attachment — Submitted filename: Response to Reviewers.docx [file pone.0299228.s005.docx]

Academic Editor and Reviewer Comments with Responses

**Academic Editor:**

**Comment**: Shorten and edit the introduction to reduce redundancy and enhance clarity

**RESPONSE**: Thank you for the suggestion, we have now shortened the introduction.

**Comment**: Include additional information about the studied population

**RESPONSE**: We have provided all of the information that we have available to use already, unfortunately this is not possible as we cannot retrospectively obtain this information as our current data is anonymized.

**Comment**: Emphasize the novelty of the study in the discussion section

**RESPONSE**: We have now emphasized the novelty of the study within this section.

**Comment**: Elaborate on the concept of "combination factors" and specify which factors mainly contributed to the avoidance of exercise. Other then clarifying what the, there is no further specification that we can give that it not already elucidated within the paper already based on the data that we have.

**RESPONSE**: This has now been added.

**Reviewer 1:**

**Comment**: More information could be useful concerning the so called “disease burden” as it could affect the feasibility of physical exercise besides disease activity. Ideally, the description of the studied population should include the Montreal classification (including age of disease onset, disease location and, for Crohn’s disease, the disease behavior). I understand that given the recruitment method adopted for the research this information could not be easy to collect. At least the information about previous IBD surgeries (yes/no, and if yes how many time, is a stoma present?) could be usefully added. Other easy to collect information could be the BMI: alterations of nutritional status could also have influence on physical activity.

**RESPONSE**: Unfortunately, although this could potentially be useful information to the paper, as the data/ questionnaire responses are anonymized, it is impossible to retrospectively collect this additional information.

**Comment**: A further minor point: line 67, tofacitinib is not correctly classifiable as a biologic drug, best defined as “small molecule”.

**Response**: You are correct, we have now amended this.

**Reviewer 2:**

**Comment**: There are some revisions inside the manuscript like corrections, reduce the introduction and others must do it.

**RESPONSE**: We have now reduced the volume of the introduction section.

**Reviewer 3:**

**Comment**: In my opinion the paper is well written although different limitations are present (introduction is too long and editable, baseline characteristics of the included population are scant - for example concomitant extraintetsinal manifestations, other comorbidites etc; I would have preferred a more detailed discussion recalling available literature)

**RESPONSE**: We have now reduced the volume of the introduction section.

Unfortunately, with regards to the baseline characteristics, although this could potentially be useful information to the paper; as the data/ questionnaire responses are anonymized, it is impossible to retrospectively collect this additional information.

With great respect, we do not agree in relation to the discussion section.

My fear is the lack of novelty associated to this paper where a plenty of literature is present (and in teh discussion the real novelty is not highlighted)

**RESPONSE**: We have now highlighted the novelty of the paper to a greater extent within the discussion section at yours and other reviewers’ recommendation.

**Reviewer 4:**

**Comment**: The introduction should be shorter because of its redundancy.

**RESPONSE**: We have now reduced the volume of the introduction.

**Comment**: The readers need help understanding the meaning of another IBD condition. Please give more detailed information.

**RESPONSE**: Although Ulcerative Colitis and Crohn’s represent the overwhelming majority of cases of inflammatory bowel disease, there are other inflammatory bowel conditions such as Microscopic colitis and it is also possible for patients who suffer from both Crohn’s and Ulcerative Colitis. Therefore, the ‘other’ category was present to represent such patients. Given the 11 respondents selected this option, we feel that doing so was a valid decision. This has now been communicated within the paper.

**Comment**: What exactly are combination factors? Please describe in detail. Additionally, what combination of factors mainly contributed to the avoidance of exercise?

**RESPONSE**: The ‘combination’ of factors was created as patients were able to select multiple items on some questions, therefore, to be able to undertake the necessary chi-squared analyses which require categorical data, those who did so were categorized into a ‘combination’ category. The questions where respondents could select multiple options was already shown in Supplementary materials 3 but how the ‘combination’ variables were calculated has now been articulated within the paper.

**Comment**: What are the sources of information on patients who think aerobic exercise is beneficial for IBD disease control?

**RESPONSE**: Participants will have had access to general information about physical activity as does the public. There will be nothing specific for those with IBD.

**Comment**: Are there differences in response to the questions listed in Table 3 by age, between patients not on drug therapy and those who are, and between patients receiving biological therapy and those not receiving it?

**RESPONSE**: There were no differences as a function of age or drug therapy as was already outlined in the previous submission under the ‘Exercise perceptions, beliefs and behaviours (two-way cross-tabulation analyses’ heading.

As stated already within statistical analyses section of the paper we tested differences in responses to each question based on baseline/demographic variables, i.e. gender, age, specific IBD condition and disease activity etc, yet only statistical analyses that exhibited statistical significance are presented within the results. This was undertaken in order to improve the clarity of the results and give precedence to the statistically significant observations – the results section would be around 4-5 times longer if we wrote about all of the non-significant findings.

**Comment**: Most IBD patients seem to know little about the significance of resistance training. Why is this? Are these perceptions different by age?

**RESPONSE**: Unfortunately, as we did not specifically examine ‘why’ IBD patients know little about the importance of resistance training we only examined the questions in relation to the type of resistance training, whether respondents thought that resistance training was positive/ negative and avoidance of certain types of exercise, it is not possible to determine why this was the case. We have however, attempted to the greatest extent possible without making unsubstantiated claims why the aforementioned observation might be the case.

As per above in relation to age, the responses did not differ as a function of age or indeed any other of the baseline/ demographic parameters, in addition to what is already stated within the paper.
